# ‘Local community-based disaster management’ The transformation of religious and local wisdom values in preparation to deal with natural hazards in West Sumatra, Indonesia

**DOI:** 10.4102/jamba.v13i1.1020

**Published:** 2021-08-31

**Authors:** Syafwan Rozi, Abdul R. Ritonga, Januar Januar

**Affiliations:** 1 Department of Sociology Religion, Faculty of Ushuluddin Adab and Dakwa, State Institute for Islamic Studies (IAIN), Bukittinggi, West Sumatra, Indonesia; 2 Department of Islamic Scripture, Faculty of Ushuluddin Adab and Dakwa, State Institute for Islamic Studies (IAIN), Bukittinggi, West Sumatra, Indonesia

**Keywords:** community-based disaster management (CBDM), local wisdom, religious, local community, natural disaster, transformation

## Abstract

Community based disaster management (CBDM) empowers communities to be actively engaged and be pro-active in disaster management. The involvement of the community is one of the keys to success in disaster management, especially considering the values embraced by the community itself such as religious values and local wisdom. This study aimed to create and implement a CBDM model based on religious and local wisdom of Minangkabau people in West Sumatra, Indonesia. By using Research and Development (R&D) design with a generic adaptive model of Creswell from Gall and Borg, the researchers created, implemented and evaluated a CBDM model based on religion and local wisdom of some of the nagari (villages) in West Sumatra that are prone to natural hazards. The findings of the research at the model formulation stage have been conducted, elaborated and developed incorporating the local values such as rituals and ceremonies, together with customary laws that govern behaviour, and strengthen social cohesion in order to be more applicative, practical and effective in disaster management. Furthermore, quantitative tests were conducted so that this model had the value of practicality and effectiveness to be applied to other communities in any disaster area.

## Introduction

West Sumatra is one of the disaster-prone areas in Indonesia. It is because the geographical and geological location of this area is close to the Australian plate; and it has 31 mountains spread across seven districts which at any time can cause earthquakes and other disasters (National Board for Disaster Management Team 2009). From 2005 until 2009, the West Sumatra province encountered many devastating disasters (Zed 2012). In 2005, Padang was struck by a 5.5 Richter Scale-earthquake. Then, on 06 March 2007 a tectonic earthquake measuring 6.3 on the Richter Scale occurred in West Sumatra because of the plate movement of Mount Tandikat which destroyed 45 000 houses and killed 70 people (Ismail 2007). The flash flood (*Galodo* in Minang Language) in Jorong Sasai Kandang (Saskan) Malalak, Agam Regency, 10 November 2008 added to the varied natural hazards that hit this province (Anonymous 2018). Finally, in November 2009, Padang and Padang Pariaman were hit by a 7.9 on the Richter Scale-earthquake, and were violently destroyed (Zed 2012).

Studies on the dynamics of the natural disaster management in West Sumatra have been widely carried out and documented (Efendi 2007, Danhas 2011, Sunarti 2009, Damsar 2011 and Zed 2012). Especially, Danhas (2011) concluded that the disaster management in this area has not been managed and coordinated well. Even the involvement of the community with its cultural values has not been utilised maximally. This is contrary to the principles adopted by this region through a strong social system and structure, plus the religious system that is held firmly by the people.

The community with its local potential in managing disasters is in a very decisive position in addition to government functions (ADPC 2006). Anthropologically, society is not an independent entity. There are a set of values, morals and institutions that influence individual behaviour in a society. Knowledge and belief in society include a set of concepts, values, category systems, methods and theories that are used selectively in dealing with and interacting with the surrounding environment (Woodhead, Heelas & Martin 2013). Community-based disaster management (CBDM) is a model currently applied to disaster management (Maskrey 2011). The mechanism of CBDM is not only about the application of mitigation actions at the community level, but also about paradigm change of the role of society from the object – the sufferer to the subject who plays a dynamic role (Islam 2013). Consequently, this model seeks to develop and implement strategies that are locally appropriate and being ‘owned’ for disaster management.

In particular, this study wanted to uncover the application of religious values and local wisdom of Minangkabau indigenous community, a majority ethnic group in West Sumatra, in disaster management. In principle, the Minangkabau people have strong local wisdom through religion-social system, government system, and local wisdom values in facing life and even disasters (Efendi 2007). The religious values and local wisdom of Minangkabau people in disaster management are: *Tambo* (proverbial saying), a philosophy of life of Minangkabau about disaster preparedness and management; *Badoncek* (local social security system in natural disaster anticipation management); and *Rumah Gadang* (the house architecture and local layout in the anticipation of natural hazards) (Rozi 2017). The Minangkabau local values in managing disaster conceptually also have developed since hundreds of years. It’s just that these local values have not been structured and applied in today’s popular CBDM system.

In this study, we created and implemented a CBDM model, with a focus on designing a disaster management model, implementing and evaluating the application of that model for Minangkabau people of West Sumatra. This model was formulated with the actualisation and transformation of the religious values and local wisdom of the *nagari* community in several disaster-prone areas in West Sumatra.

## Literature review

In general, disaster studies have focused a lot on community involvement in disaster management such as Oktari et al. (2018), Ikeda and Nagasaka (2011), Cronjé, Reyneke and Van Wyk (2013), and Aka et al. (2017). Furthermore, the more specific involvement of local communities with their wisdom in disaster management has been found by Berse, Bendimerad and Asami (2011), Rapeli et al. (2017), Rozi (2017), and Kusumasari and Alam (2012). The use of community participation with local values has successfully proved that the severity of the impact of disasters is because of the weakness of top-down disaster management strategies that ignore local potential resources and capacities (Kapiarsa & Sariffuddin 2018). Even in some cases, this pattern of disaster management can increase people’s vulnerability to disaster risk. It is a pattern of disaster management carried out by community members in an organised manner before, during and after a disaster using the resources they have (Kafle 2004). This is considered to be able to prevent, reduce, avoid and even recover from the impact of disasters by utilising local potential and values.

Community-based disaster management as a structured model in involving the community specifically has been studied by researchers and their teams such as Pribadi et al. (2011), ADPC (2006), and Kafle (2004). The interesting part in CBDM is its objectives which are to (1) reduce the vulnerability and increase the capacity of groups and communities to cope with, prevent or minimise loss and damage to life, property and environment, (2) minimise human suffering, and (3) accelerate recovery, (Maskrey 2011). According to (Islam 2013), the vulnerability reduction process aims to negotiate resources and support from local and central governments for risk management actions on all fronts. Thus, CDBM assists communities in building their local potential to face disaster emergencies.

However, to build local participation and potential in disaster management is incomplete if it does not utilise religious values and local wisdom or local indigenous knowledge of the community. The studies conducted by Kelman, Mercer and Gaillard (2012), Syafwina (2014), Hiwasaki, Luna and Marçal (2015) and Iloka (2016) have emphasised that local indigenous knowledge based on traditional or religious beliefs, such as rituals and ceremonies, together with customary laws that govern behaviour, and strengthen social cohesion, help communities to face and respond to disaster risk and the impacts of climate change and climate-related hazards in a better way.

In addition, the failure in understanding and applying religion and local wisdom also results in the unsustainability of disaster management at the grassroots level. Such models are generally incompatible with the socio-cultural conditions of the community itself. This was because disaster management agendas were not born from the awareness and priorities of the community itself. In several studies of disaster history and disaster anthropology, there were many interesting cases that were worth studying such as how the institutionalisation of knowledge about mitigation has been hundreds of years old, and has been used as a community tradition (Syafwina 2014). It was just that the values have not been organised and did not accommodate the current needs.

Therefore, CBDM based on religion and local wisdom values was expected to be able to exploit the local potential in the process of disaster management. Although this model was often considered similar to CBDM, the difference was in an approach that encouraged grassroots communities. As a result, it was necessary to study and develop the application of CBDM model based on religious values and local wisdom so that this community participation model was more contextual and the local values are more applicative.

## Methods

The research method used was a Research and Development (R&D) with a generic adaptive model of Borg and Gall (1989) and Mc Millan and Schumacher (2001). This model was later developed by Creswell (2008) through the following steps: (1) a preliminary study, (2) formulation of a conceptual model, (3) validation of a conceptual model, (4) reflection and revision of a conceptual model, (5) limited trial, (6) revision of the model, (7) extensive trial, (8) analysis and discussion, (9) conclusions and recommendations. However, because of the limitation and effectiveness of the presentation of this article, the research method used only four steps that is, formulation of a conceptual product or model, limited trials (practicality tests), extensive trials (effectiveness tests) as well as analysis and discussion.

The research implementation of the CDBM model was conducted on five communities of disaster victims in West Sumatra as informants and target groups. This study conducted two types of tests, namely practicality test and effectiveness test. In practicality tests, the researchers used structured questionnaires as data collection techniques, which were distributed to five Minangkabau indigenous communities as the owners of religious values and local wisdom. The sampling study consisted of 25 respondents who were selected by purposive sampling. The data analysis in this practicality test used the average formula to test whether the results were practical or not.

As for the effectiveness test, this study used two data collection techniques, namely questionnaires and interviews. The questionnaire was used for quasi-experimental quantitative techniques by testing this model on two groups: experiment group (trial group) and control group (comparison group or model pilot). The informants consist of 41 people for the experimental group and 39 people for the control group. This data was then analysed using T-Test to measure which hypothesis was rejected and accepted. Furthermore, the researchers also used interview as data collection technique for the effectiveness test. This was conducted to obtain the qualitative data on the response and opinion of informants whether this model was effective or not which cannot be obtained by using questionnaires. The qualitative techniques were also used to analyse and compare with quantitative data that has been done.

## Result and discussion

### Conceptual model

Nowadays, it is rare to find studies that seriously discuss about the local CBDM model in West Sumatra, except for a few reports and the implementation of general models from organisations such as PMI, BNPB, BPBD (regional agency for disaster countermeasure) as well as international donor agencies working in West Sumatra. Meanwhile, this region which holds firmly its traditional and religious order is losing on the optimal implementation of disaster management by not utilising human resources with the values of its local wisdom.

The CBDM model through the transformation of *adat* (tradition) and religious values is designed to answer the problems of disaster management. In principle, the optimal implementation should mobilise the resources that are owned and controlled and is an internal part of the daily life of the community. This understanding is important because the grassroots people who face threats are not powerless as construed by the technocrats.

Based on the theoretical concepts regarding CBDM, the researchers formulated a conceptual model at the research stage. This conceptual model was born based on an in-depth study of religious values and local wisdom of the Minangkabau community in disaster management. These rich values which are inherited from generation to generation are identified, modified and structured into the operational steps and can be applied as the model of operational steps in the disaster management system in West Sumatra (see Table 1).

**TABLE 1:** Local wisdom in West Sumatra local community.

Variable	Sub-variables	Indicators
The transformation of local wisdom values in natural hazards management in West Sumatra	*Tambo* (proverbial saying and philosophy of life of Minangkabau about disaster preparedness and management)	The philosophical content of *Tambo*
		The purpose and meaning
		The operational target
	*Badoncek* (local social security system in natural disaster anticipation management)	The meaning and purpose
		The steps
		The impacts
	*Rumah Gadang* (the house architecture and local layout in the anticipation of natural hazards)	The meaning and purpose
		The steps
		The impacts

Source: Rozi, S., 2017, ‘Local wisdom and natural disaster in West Sumatera’, *el Harakah* 19(1), 1–20. https://doi.org/10.18860/el.v19i1.3952

The three local values, based on Table 1, represented the number of local wisdoms of the Minangkabau people in addition to the social system and *adat* (culture) kinship as the adhesive for the social relations of indigenous people in disaster management. These local values were then transformed into three stages based on disaster management platforms such as prevention, emergency response, and post-disaster recovery planning.

Furthermore, this model was developed in a model module format in the form of stages and steps for implementing disaster management based on the religion and local wisdom. This implementation step based on the community participation had a sequence of learning that began with identifying, gathering information, reviewing problem solving, proposing problem solving, displaying the cycle of community empowerment.

### The practicality test of the disaster management model based on religion and local wisdom

A research with R&D method requires the researchers to develop a valid and reliable model. This means that the model developed must be truly capable of solving problems encountered whenever and wherever the model is implemented. The general and applicable model that has been formulated in the local Minangkabau case should be applicable to local communities throughout Indonesia. Generally, people have common values and principles that have similarities, but the terms and the operational mechanism are different.

To answer this challenge, in the R&D method phase, the researchers are required to be able to try out the model they have developed in other groups in different situations and conditions. A limited trial or a practicality test is trial conducted on a small group of people to prove whether the developed model is effective enough to overcome the problem. If the limited trial obtains the results that the developed model is concluded to be practical in overcoming problems or achieving certain goals, the next step is planning to carry out an extensive trial. The assumptions from extensive trial prove that the resulted model can be implemented for anyone outside the limited trial group. In addition, the extensive trial is used to improve the practices that were imperfect when the limited trial was conducted. A model would be judged to have a high level of reliability when the results are consistently seen from the perspective of effectiveness between the limited trial and the extensive trial.

The analysis of practicality test for the model of disaster management based on local wisdom was one of the trials conducted to test whether this pre-determined model had practicality value. This trial was determined from the results of a simple questionnaire that was used and understood in the implementation of disaster management. The general target of the question was whether this model was easy to be used and understood by the facilitators and community members (Borg & Gall 1989):

$$R=\frac{\sum_{i=1}^{n}V_{i}}{n}$$

Where:

*R* = the average of the evaluation results from the experts or validators

*V*_i_ = the score of the 1st validator assessment results

*n* = number of validators

The average results of all aspects of the practicality of the model are analysed using the following criteria:

- If the average is > 3.20, it is categorised as very practical;
- If 2.40 < average ≤ 3.20, it is categorised as practical;
- If 1.60 < average ≤ 2.40, it is considered quite practical;
- If 0.80 < average ≤ 1.60 is categorised as less practical;
- If the average is ≤ 0.80, it is categorised as not practical.

For the development of this analysis, a model is said to be practical if the average value of the practitioner has enough practical value. The prototype practicality questionnaire is described by data frequency analysis techniques using the formula in Equation 2 (Creswell 2008):

$$\text{Practicality=}\frac{\text{Average Score}}{\text{Maximum Score}}\times 100\%$$

Table 2 reports the result of the practicality test conducted on the results received from the questionnaire distributed to 25 respondents by submitting 10 statements using the option of agreeing and disagreeing. The respondents consisted of practitioners, disaster activists, observers and users at the community level.

**TABLE 2:** The results of practicality test on local wisdom-based disaster management.

Number	Statements	Amount	Average		Category
			*n*	%	
1	The developed module has elements that can attract the attention of the community.	23	3.8	92	VP
2	The use of images or matrices can help the community in understanding the module.	21	3.5	84	VP
3	The simulation activities in the module can facilitate the community in knowing religious, *adat* and cultural values that exist in disaster management.	20	3.3	80	VP
4	The need assessment activities in the module can facilitate the community in understanding the module.	23	3.8	92	VP
5	The planning activities in the module can facilitate the community in understanding the module.	23	3.8	92	VP
6	The activities and participation cycles of the community in the module are easy to understand	23	3.8	92	VP
7	The developed module contains religious values that are practical and operational in disaster management.	21	3.5	84	VP
8	The developed module contains the values of local wisdom that are practical and operational in disaster management.	23	3.8	92	VP
9	The use of module can help the facilitator to improve the community capacity.	20	3.3	80	VP
10	The use of language, font size and font in the module makes it easier for the facilitator and the community.	21	3.5	84	VP

**-**	**Total**	**218**	**3.61**	**87.2**	**VP**

VP, very practical.

Based on Table 2, it was concluded that the implementation of disaster management model based on religion and local wisdom was very practical with a total score of 218, with an average of 3.61, equivalent to 87.2% of the very practical category. Thus, the community module can be declared eligible to be used.

### The effectiveness test of disaster management model based on religion and local wisdom

After the practicality test, the next trial was the effectiveness test which was to test the effectiveness of a resulting model. In this case, qualitative and quantitative data were used. The qualitative data such as observations and interviews (in this case such as community reactions, community learning activities, activities and creativity) were analysed qualitatively. The quantitative data were obtained using quasi-experiment model by comparing test results in the experimental group with the control group. The questionnaires were distributed to 41 respondents of the experiment group and 39 respondents of the control group. Furthermore, the results of this test were analysed by using T-test to test hypotheses H1 and H0. The true status of H1 if T-count > T-table, shows there is a significant difference. Or the true status of H0 if T-count < T-table, shows there is no significant difference.

To test the effectiveness, the experiment was carried out by conducting the implementation of disaster management model based on the religion and local wisdom in the experimental group and compared it with the control community. The experiment was carried out in two KSB (*Komunitas Siaga Bencana*/Disaster Preparedness Community) *Nagari* (village), West Sumatra. The KSB Nagari Ketaping was chosen as the experimental group and the KSB Nagari Balingka became the control group. It was because the two groups had the same conditions and they were randomly chosen in which one became the experiment group and the other the control group. Table 3 presents the analysis of quantitative data from the experimental group as well as the control group.

**TABLE 3:** The descriptive statistics of the experiment group and the control group.

Variable	*N*	Minimum	Maximum	Mean	Standard deviation
Pre-test experiment	41	3	15	7.56	3.171
Post-test experiment	41	10	20	14.37	2.395
Pre-test control	39	3	12	7.21	2.386
Post-test control	39	9	16	11.21	2.226
Valid *N* (listwise)	39	-	-	-	-


Based on Table 3, it was found that there was an increase in the average score of the experimental group from 7.56 at the pre-test to 14.37 at the post-test level. An increase also occurred in the control group from 7.21 in the pre-test level to 11.21 in the post-test level.

The next test was the analysis compare means paired-sample T-Test. This test was conducted to test the significant difference in the effectiveness of the model in the post-test of the experimental group and the post-test of the control group. However, before testing the analysis, normality test is needed to prove whether the variables from the data obtained are normal. Thus, each variable is first tested for normality by using the Kolmogorov-Smirnov Test statistical test with the help of the SPSS Version 18.00 programme. With a significant level of 0.05, the data is declared normally distributed if the significance is greater than 0.05.

Table 4 reports the post-test experimental group normality test. It can be seen from Table 4 that the test results show a significant value of Kolmogorov-Smirnov normality, namely (1.248) > from alpha (0.05) so that the data is normally distributed. Furthermore, the post-test control group normality test is presented in Table 5.

**TABLE 4:** One-sample Kolmogorov-Smirnov Test.

Item	Variable	Value
Post-test experiment group	*N*	41
Normal parameters	Mean	14.37
	Standard deviation	2.395
Most extreme differences	Absolute	0.195
	Positive	0.195
	Negative	−0.98
Kolmogorov-Smirnov Z†	-	1.248
Asymp. significance (2-tailed)	-	0.089

† , Test distribution is normal.

**TABLE 5:** One-Sample Kolmogorov-Smirnov Test.

Item	Variable	Value
Post-test control group	*N*	39
Normal parameters	Mean	11.21
	Standard deviation	2.226
Most extreme differences	Absolute	0.193
	Positive	0.193
	Negative	−0.161
Kolmogorov-Smirnov Z†	-	1.205
Asymp. Significance (2-tailed)	-	0.109

† , Test distribution is normal.

The test results as presented in Table 5 show that the Kolmogorov-Smirnov normality value is significant (1.205) > from alpha (0.05) so that the data is normally distributed. After all variables are declared normal, then the next step is the analysis compare means paired-sample T-Test. The result of the analysis compare means paired-sample T-test are presented in Table 6.

**TABLE 6:** The result of analysis compare means paired-sample T-test.

Groups	T_count_	*df*	T_table_	Significance	Description
Post-test experimental group: post-test control group	6.039	78	2.64	0.01 (1%)	There was a very significant difference

*df*, degrees of freedom.

Based on the result in Table 6, the difference of the test result between the post-test in the experiment group and post-test in the control group was T-count of 6.039. The significant level of 0.01 (1%) and a degree of freedom (*df*) of 78 was obtained T-table of 2.64. Thus, T-count (6.039) > T-table (2.64), and therefore the H1 hypothesis was accepted and H0 was rejected. There was a very significant difference on the effectiveness of the model in the post-test of the experimental group and the post-test of the control group.

In addition, the research with the use of qualitative method in several stages of data collection through Focus Group Discussions (FGD), in-depth interviews and observations found several problems and phenomena in the implementation of religious and local wisdom value in disaster management model based on the religion and local wisdom including social construction, social security and community participation management. Based on the observations conducted by the researchers in several KSBs (*Komunitas Siaga Bencana*/disaster preparedness community) in West Sumatra, it seemed that the community’s ability to cope with disasters was still low. This was in line with the findings in quantitative analysis. Although it was considered effective, the effectiveness level was considered not strong enough. This conclusion was supported by interview data on the response and perception of the local community about the application of this model. This can be seen from the low level of knowledge and participation of the community regarding disasters and how disaster management was carried out. Many KSBs of the *nagari* (village) in West Sumatra at the district and city level were generally inactive, while *Basnas* (*zakat* collection agency of Indonesia) was generally still at the level of structures that were not yet active and functioning.

## Analysis and discussion

The purpose of the research on designing and implementing the model of ‘community-based disaster management’ based on religious values and local wisdom was to reconstruct and increase the capacity of local communities with local values through disaster management mechanisms. Ideally, this mechanism was carried out actively by the elements of society such as families, social organisations and surrounding communities before, during and after a disaster.

However, the application of religious values and local wisdom must be done in a systematical and structured way. The rich local values owned by the local community were still traditional and indigenous, and were even claimed by many circles of people as values that are old and not updated with the current developments. Therefore, adaptation and modification of these values were needed in order to be accommodating and flexible in answering the needs of the community in disaster management. Therefore, a systemic model was needed to make these values more applicative in disaster management.

The study of the implementation of the ‘community based disaster management’ model based on religious values and local wisdom has answered the issue of the application of these local values. At the stage of model formulation, it was necessary to deepen the local values such as rituals and ceremonies, together with customary laws that govern behaviour, and strengthen social cohesion. These traditional values must be elaborated and developed by determining the indicators and efforts to be more applicative, practical and effective in disaster management.

To know whether this model has practical value and can be applied to different communities, it was necessary to test the practicality of the model. Furthermore, to know whether this model was effective in addressing community problems, the effectiveness test was also required. In practicality term, this model was practical. However, to achieve an effective level, it takes a long time and this model needs to be continuously tested and developed.

Finally, the model was considered to be good as it was able to facilitate and stimulate the potential of disaster preparedness groups so that it became a competency that can be used to build its environment in the global era. The model must be able to produce a disaster community that is creative and innovative and able to lift its potential. In addition, the model of disaster management based on religion and local wisdom also accommodates elements in community learning, even of course by considering existing models issued by relevant agencies such as national and international disaster agencies.

## Conclusion

After conducting the research in implementing the ‘community-based disaster management’ model through the transformation of religious values and local wisdom in disaster preparedness in West Sumatra it was concluded that: (1) The local wisdom values of the Minangkabau community were local or indigenous knowledge in the form of the knowledge of the indigenous people who lived in certain geographical locations, which had different cultural and belief systems from the modern intellectual world knowledge system. It was a collection of knowledge created by a group of people from generation to generation that lived united and in harmony with nature. (2) The formulation of an *adat* (culture) and religious-based disaster management model in West Sumatra was a participatory and planned management model. The CBDM model through the transformation of *adat* and religious values provided the answers that included several principles such as efficiency because of the low transaction costs. It was because there were maximum local inputs and minimum external inputs. (3) After the model was determined, practicality test was carried out to know whether this model had a practical level. Based on the results of this trial, it explained that the pre-determined model was at a practical level or can be applied even with some revisions. (4). Next, the effectiveness test was conducted to find out whether the model was effective to be implemented. This test was conducted in the form of a questionnaire given to the experimental group and the control group after the implementation of the created model and the conventional model. Based on these test, it was found that this model had a high level of effectiveness to be implemented. However, at all levels of the test there were a number of records that needed revision and improvement before the model was finalised to become the final model.

Therefore, the ‘community-based disaster management’ model through the transformation of religious values and local wisdom enabled the community to be more pro-active in the problem of disaster. In principle, people had better knowledge of disasters than the state because they were the ones who knew better about the real conditions of their respective environments Kelman et al. (2012), Hiwasaki et al. (2015) and Iloka (2016). Therefore, this model provided space and a model of how people and local communities with their wisdom values were empowered in overcoming their problems regarding natural hazards.
